# RET gene fusion and emergent Selpercatinib resistance in a calcitonin-rich neuroendocrine carcinoma: a case report

**DOI:** 10.3389/fonc.2024.1360492

**Published:** 2024-02-14

**Authors:** Reza Pishdad, Peter B. Illei, Christopher D. Gocke, Douglas W. Ball

**Affiliations:** ^1^ Division of Endocrinology, Diabetes & Metabolism, Johns Hopkins University School of Medicine, Baltimore, MD, United States; ^2^ Division of Endocrinology, Diabetes & Metabolism, Massachusetts General Hospital, Harvard Medical School, Boston, MA, United States; ^3^ Department of Pathology, Johns Hopkins University School of Medicine, Baltimore, MD, United States; ^4^ Department of Oncology, Johns Hopkins University School of Medicine, Baltimore, MD, United States

**Keywords:** RET, Selpercatinib resistance, medullary thyroid cancer, MTC, RAS, neuroendocrine lung cancer, Ulixertinib, KIF5B-RET

## Abstract

Metastatic lung neuroendocrine carcinomas provide diagnostic challenges in identifying the cell of origin. High level calcitonin expression is not pathognomonic for medullary thyroid cancer. Tumor mutation analysis may provide essential clues regarding tissue origin and treatment targets. Oncogenic RET gene fusions have been identified in non-small cell lung cancer and non-medullary thyroid cancers, whereas RET point mutations are the key genetic finding in both inherited and sporadic MTC. Patients who receive radiation for the treatment of other cancers have an increased risk of developing a second malignancy, including a neuroendocrine carcinoma. Herein, we present a case of calcitonin-rich neuroendocrine carcinoma emerging on a background of prior radiation and chemotherapy for the treatment of Hodgkin’s disease. Identification of a RET gene rearrangement (KIF5B-RET) led to initial successful treatment with selpercatinib, with eventual resistance associated with an activating mutation involving the MEK1 protein (MAP2K1 p. E102-I103 del) that led to relapse and progression of the disease.

## Introduction

Oncogenic *RET* gene fusions have been detected in non-small cell lung cancer and non-medullary thyroid cancers. Conversely, *RET* point mutations represent the principal genetic anomaly in both hereditary and sporadic cases of medullary thyroid cancer (MTC) (1, 2). A *RET* gene translocation mechanism is exceedingly rare in MTC and has been described to date in only one case (3).

The identification of a *RET* gene fusion serves as a specific target for the utilization of selective *RET* inhibitor medications. Selpercatinib is an FDA-approved oral tyrosine kinase inhibitor (TKI) that selectively targets *RET* mutated tumors.

Drilon and colleagues’ assessment of Selpercatinib’s effectiveness in *RET* fusion-positive lung cancer demonstrated rapid and enduring anti-tumor efficacy. The outcomes observed in patients treated with Selpercatinib surpassed prior achievements attained with multikinase inhibitors. Within their investigation, 64% of patients administered Selpercatinib exhibited a measurable and long-lasting positive response. Notably, among individuals who had not received prior treatment, 85% displayed a response, suggesting a durable effect.

In contrast to multikinase inhibitors, Selpercatinib is primarily linked to mild toxic effects, predominantly of low severity. This is attributed to its *RET*-selective nature, which results in minimal off-target activity. The most common grade 3 adverse events observed were reversible through adjustments in dosage, indicating the feasibility of long-term treatment and suggesting the potential for sustained therapy (4).

Herein, we describe an unusual patient with calcitonin-rich lung neuroendocrine tumor indeterminate for MTC versus adenocarcinoma with neuroendocrine features, with a *KIF5B-RET* fusion mutation, suggesting a primary lung etiology, who demonstrated initial excellent response to targeted therapy, but occurrence of a new activating mutation in the *MAP2K1* gene led to relapse and progression of the disease.

## Case presentation

**Figure 1:**
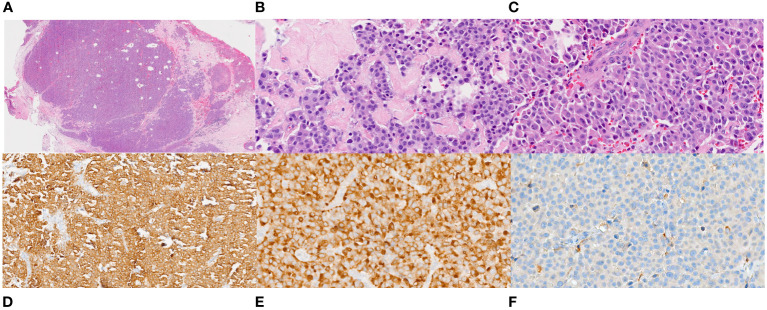
Histopathology of the VATS biopsy of the mediastinal node. **(A)**. Low power (20X) view of hematoxylin and eosin-stained section of 4R lymph node showing a lymph node entirely replaced by an epithelioid neoplasm with areas of dense fibrosis and fibrous bands. **(B, C)**. High power magnification (400X) of the tumor showing relatively uniform tumor cells with abundant dense eosinophilic cytoplasm and round to oval nuclei some that are located at one side of the cells (plasmacytoid appearance). The nuclei have fine chromatin and no or small nucleoli. **(D, E)**. Immunostains for cytokeratin (200X) and calcitonin (400X) are diffusely positive in the tumor cells. **(F)**. An immunostain for PAX-8 (400X) is negative.

**Figure 2:**
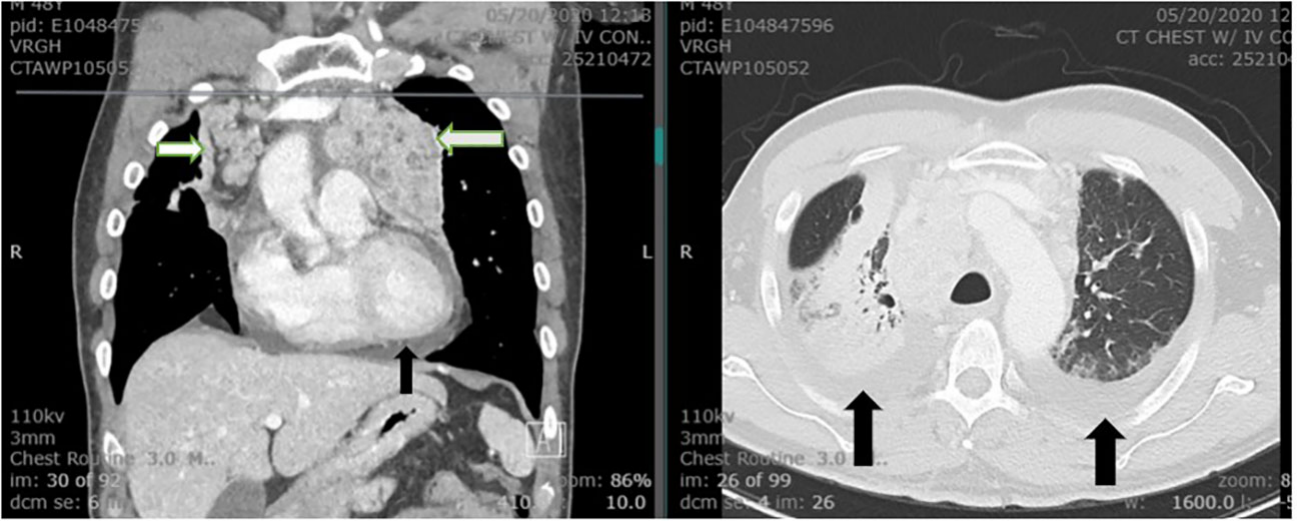
The CT of chest demonstrates extensive bilateral hilar and mediastinal lymphadenopathy (white arrows). Bilateral pleural effusion and moderate pericardial effusion are noted (black arrows). The visualized portion of the thyroid gland is normal.

A 50-year-old male presented to our institution with cough, fatigue, heat intolerance, and flushing. His only notable medical history was stage IIIBS Hodgkin lymphoma, nodular sclerosis type, which was treated with Adriamycin, Bleomycin, Vinblastine, and Dacarbazine (ABVD regimen) for 6 cycles. This was followed by consolidative radiotherapy with a course of 30 Gy radiation distributed over 17 treatment sessions targeting bulky mediastinal nodes, which commenced 4-6 weeks after his last cycle of chemotherapy. The patient had never smoked cigarettes, nor did he have any family history of cancer. He remained in remission for approximately 6 years. The CT chest scan identified newly developed bulky mediastinal and hilar adenopathy along with enlarged cervical nodes. The VATS biopsy of a mediastinal node disclosed a metastatic neuroendocrine neoplasm exhibiting plasmacytoid features, focal necrosis, and a mitotic rate of up to 10 mitoses per 2 mm^2^. The tumor cells demonstrated immunoreactivity for calcitonin, CEA, synaptophysin, and cytokeratin AE 1/3 and CAM 5.2, while being negative for S100, HMB45, SOX-10, MUM-1, CD20, TTF1, and PAX-8 (**Figure 1**). The differential diagnosis included MTC or metastatic atypical carcinoid tumor. Next Generation Sequencing (NGS) using Integrated DNA Technology yielded negative results, including *RET* and *NRAS* point mutations. Subsequent testing for rearrangements with an Actionable Fusion Panel using RNA extracts showed a *KIF5B-RET* gene fusion, an oncogene commonly identified in lung adenocarcinoma. The analysis for fusion yielded negative results for *NTRAK 1/2/3* and *BRAF.* Immunochemistry results were positive for *MLH1, MSH2, MSH6, PMS2*, and negative for *PD-L1 (SP142).* The Tumor Mutational Burden (TMB) was 3, indicating a low mutation burden. Microsatellite Instability (MSI) results were stable. Biochemical findings included a calcitonin level of 16,033 pg/mL (normal < 10), CEA of 6.9 ng/mL (normal < 4.7), and chromogranin A of 146.6 ng/mL (normal < 101.16). ACTH and cortisol levels were normal. Neck ultrasound revealed a calcified 5 mm right upper pole thyroid nodule and extensive bilateral cervical lymph nodes. A provisional diagnosis of metastatic MTC was made, and treatment with vandetanib was initiated. Two months later, he reported worsening orthopnea, diarrhea, and weight loss. The CT chest revealed marked worsening with the enlargement of mediastinal, hilar, and supraclavicular adenopathy, accompanied by new moderate-sized pericardial and pleural effusion (**Figure 2**). No intrabronchial lesions were reported. Vandetanib was discontinued, and selpercatinib was initiated following FDA approval. He reported the prompt resolution of dyspnea and diarrhea, along with improved weight and energy. Serum calcitonin declined to < 2 pg/mL, and CEA to 1.6 ng/mL four months into treatment. Neck CT revealed marked shrinkage of lymph nodes with a persistent calcified thyroid nodule, while chest CT showed significant reduction in size of mediastinal and hilar masses, along with resolution of bronchial narrowing and right pleural effusion. Nine months after the presentation, the patient reported no chest symptoms, and both biochemical markers and imaging remained stable.

**Figure 3:**
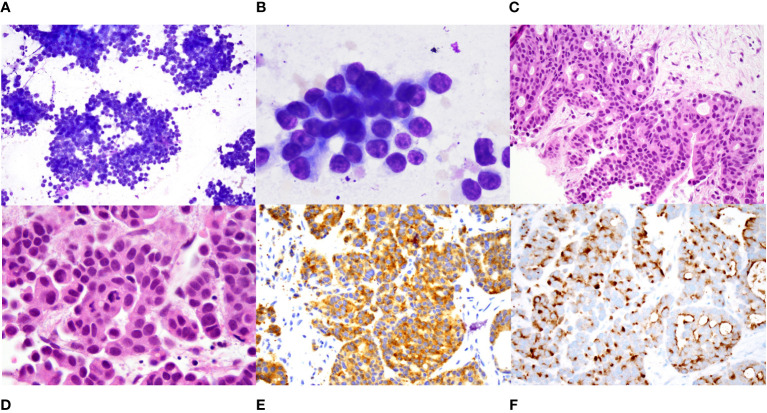
Histopathology of metastatic hepatic lesions. **(A)**. Intermediate power (100X) and **(B)**. High power (1000X) view of diff quik stained smears of a fine needle aspirate of a liver nodule showing sheets and clusters of tumor cells that have scant cytoplasm and relatively uniform round nuclei with small nucleoli. **(C)**. Intermediate (200X) and **(D)**. high power (1000X) view of hematoxylin and eosin-stained core biopsy showing nests and cords of tumor cells with focal cribriform pattern. The tumor cells have eosinophilic cytoplasm and eccentrically placed round to oval nuclei with evenly distributed smudgy chromatin, no nucleoli, and scattered mitoses. **(E)**. Immunostain for synaptophysin shows diffuse positivity (400X). **(F)**. Immunostain for calcitonin shows diffuse perinuclear and cell surface staining.

**Figure 4:**
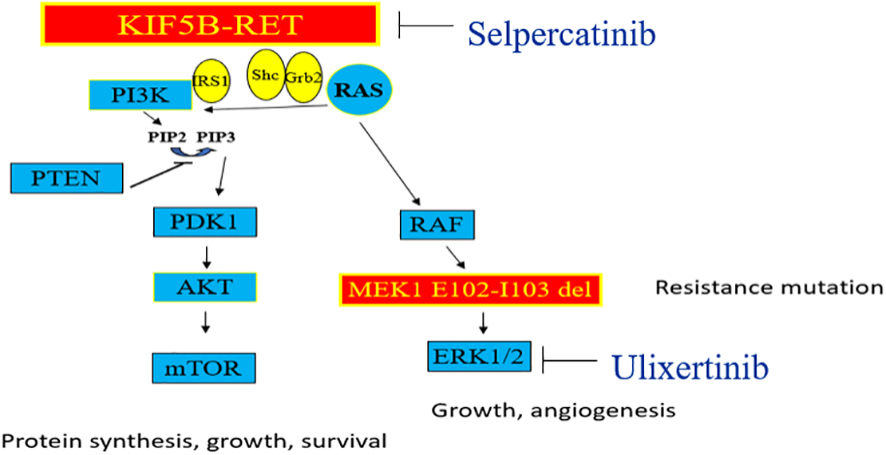
The *MAPK* pathway is seen on the right, and the *PI-3K* pathway on the left side. Genomic alterations in *RET* kinase including fusions and activating point mutations, lead to overactive *RET* signaling and uncontrolled cell growth. Selpercatinib acts to inhibit *RET* fusions whereas Ulixertinib is an investigational *ERK1/2* inhibitor which acts downstream of *MEK1* gene in *MAPK* pathway.

**Figure 5:**
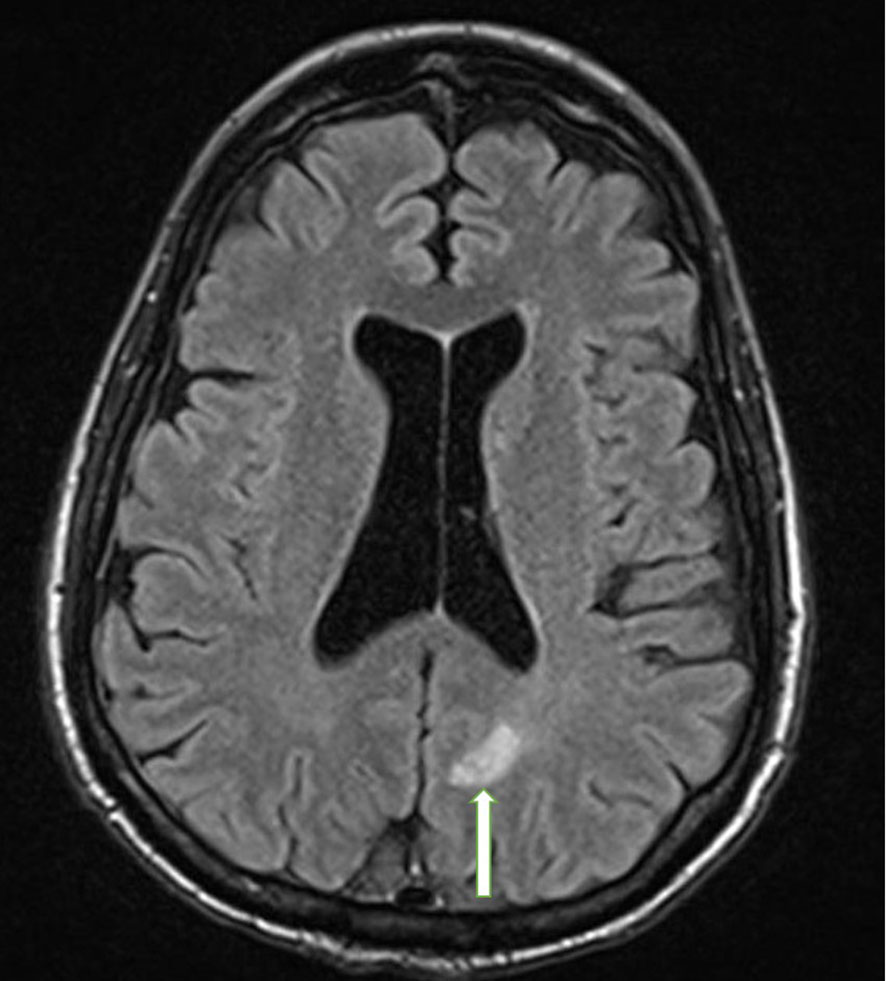
Brain MRI with and without contrast. The photograph illustrates an enhancing metastatic lesion located in the parasagittal left parietal lobe, measuring approximately 10 x 10 mm in diameter with mild diffusion restriction and mild surrounding vasogenic edema (white arrow).

Approximately 2 years after presentation, metastatic liver lesions developed. Histopathology revealed a metastatic calcitonin-positive neuroendocrine neoplasm, histologically similar to the tumor at the initial presentation, with a confirmed *KIF5B-RET* translocation. The liver lesion stained positive for calcitonin and synaptophysin, and negative for CDX2 (**Figure 3**). NGS detected a 2-amino acid in-frame deletion of *MAP2K1 p. E102-I103* that confers gain of function to (*MAP2K1) MEK1* protein, acting downstream of the *RET* fusion (**Figure 4**). The tumor mutation rate was 3.58 mutations per megabase. An MRI of the brain revealed multiple cerebral and cerebellar metastases (**Figure 5**). Unfortunately, the patient died from progression of the disease prior to salvage targeted oral chemotherapy (Ulixertinib).

## Discussion

Medullary Thyroid Cancer (MTC) represents a neuroendocrine neoplasm originating from parafollicular or C cells, constituting approximately 1-5% of the all thyroid malignancies (5). The hallmark of this particular cancer type is the distinctive expression of calcitonin and carcinoembryonic antigen (CEA).

While hypercalcitoninemia is commonly associated with MTC, it is not exclusively indicative of this condition. Modestly elevated calcitonin levels can also manifest in individuals with hypergastrinemia, hypercalcemia, renal insufficiency, neuroendocrine tumors, goiter, papillary and follicular thyroid carcinomas, as well as chronic autoimmune thyroiditis. Individuals exhibiting calcitonin levels exceeding 100 pg/mL are associated with an approximate 90-100% probability of MTC. Conversely, patients showcasing values ranging between 10 to 100 pg/mL carry a lower risk, estimated at less than 25%, for MTC (6). Elevated calcitonin levels exceeding 500 pg/mL are strongly correlated with an increased risk of either local or distant metastatic disease in individuals diagnosed with MTC (6).

Nozieres and colleagues reported the most extensive series of patients with neuroendocrine tumors exhibiting elevated secretion of calcitonin. In their neuroendocrine tumor cohort, they identified 21 individuals, constituting 12% of 176 patients, displaying serum calcitonin concentrations surpassing 100 ng/L. Their research indicated that high calcitonin levels were predominantly observed in high-grade neuroendocrine tumors originating from foregut locations, notably the pancreas and lung (7).

Patients who have undergone treatment for Hodgkin’s lymphoma may have an increased risk of developing secondary cancers, including solid tumors and other hematologic malignancies. Several factors contribute to this increased risk, including exposure to radiation therapy and certain chemotherapy agents used in the treatment of Hodgkin’s lymphoma. Regular follow-up and surveillance are crucial for individuals who have survived Hodgkin’s lymphoma to monitor and detect any potential development of secondary neoplasia. This includes the potential emergence of a neuroendocrine carcinoma (NEC) capable of secreting substances such as calcitonin or CEA (8). This situation introduces a diagnostic challenge in this patient whether the primary malignancy is an MTC or a lung cancer with NE features.

Rearrangements involving the *RET* gene with the N-terminal portion of the kinesin family member 5B gene (KIF5B) have been detected in a subgroup of lung adenocarcinomas, as well as in neuroendocrine neoplasms of the lung, such as typical and atypical carcinoid tumors, and large cell neuroendocrine carcinoma (9). In lung adenocarcinoma, researchers have described 12 distinct *RET* fusions, with the fusion involving KIF5B being the most frequently observed. This particular fusion exhibits an overall prevalence of approximately 2% among cases of lung adenocarcinoma (1). The fusion between *KIF5B* and *RET* results in the abnormal activation of *RET*, which has prompted the initiation of targeted therapy trials specifically designed for this disease. Selpercatinib has gained authorization from the US Food and Drug Administration (FDA) for managing non-small cell lung cancer and thyroid cancer harboring *RET* fusions, *RET*-mutant MTC, and solid tumor cases exhibiting *RET* gene fusions with limited treatment options.

Although *RET* point mutation is the primary driver of tumorigenesis in MTC, translocations of *RET* gene is exceedingly rare and described in only one case (3). In our patient, the identification of the *KIF5B-RET* fusion offers molecular evidence supporting a lung-based origin despite inconclusive findings from immunopathology and serum calcitonin assessments. The detection of a *RET* translocation presents a viable target for therapies involving *RET* tyrosine kinase inhibitors (TKIs).

In cases of Papillary Thyroid Carcinoma (PTC), *RET* fusions are frequently observed in pediatric patients with documented exposure to radiation, which includes therapeutic radiation treatments and nuclear accidents. However, it’s important to note that radiation exposure is not recognized as a known risk factor for the development of MTC (10).

The emergence of resistance to Selpercatinib poses a growing challenge in cases of *RET-*mutant lung and thyroid cancer. In the pivotal trial led by Wirth and colleagues, the effectiveness of Selpercatinib was evaluated in patients with *RET-*mutant MTC who had prior treatment exposure to vandetanib, cabozantinib, or a combination of both medications. The study reported a 69% objective response rate among patients, with a 95% confidence interval between 55 to 81. Of these, 9% achieved a complete response, while 60% experienced a partial response. At the one-year mark, 86% of the responses (95% CI, 67 to 95) were still ongoing, and 82% (95% CI, 69 to 90) of all patients remained free from disease progression. Regarding biochemical responses, 91% of patients (95% CI, 80 to 97) exhibited a response in calcitonin levels, while 66% (95% CI, 52 to 79) showed a response in CEA levels (11).

The protein encoded by the *MAP2K1* (*MEK1*) gene functions as a serine-threonine kinase, contributing to various cellular activities including proliferation, differentiation, transcriptional regulation, and development through the phosphorylation of mitogen-activated protein kinase (12). An activating mutation in this gene typically arises downstream of a *RET* mutation in the *MAPK* pathway (**Figure 4**), potentially offering an alternative pathway for Selpercatinib inhibition of *RET*.

Rosen and colleagues have delineated various mechanisms of resistance to *RET* inhibitors in both lung and thyroid cancer. The resistance observed with Selpercatinib is primarily due to reactivation of the *MAPK* pathway, which occurs through multiple distinct routes: solvent front mutations in the *RET* gene itself, amplifications in *MET*, and additional activating mutations further downstream in the *MAPK* pathway. In their research encompassing combined treatment groups for thyroid and lung cancer, 18 patients developed tumor resistance during the study and underwent comprehensive characterization. Among these, three out of the 18 patients with identified resistance exhibited secondary *RET* mutations, while seven out of 18 individuals demonstrated bypass mutations, such as *KRAS*, *NRAS*, *BRAF* mutations, or amplification of *MET* or *FGFR1* (13). Our patient had equivocal evidence of amplification of *FGFR4*, judged not to be significant in this case.

The *MEK1* activating mutation identified in our patient, *MAP2K1 p. E102-I103*, has been previously described by Gao and colleagues as a resistance mutation to *EGFR* inhibitors in NSCLC (13). In this study, deletion of *E102-I103* was linked to the constant activation of *MEK1* irrespective of upstream *MAPK* signaling. This mutated form of *MEK1* displayed resistance to allosteric *MEK* inhibitors like trametinib (13).

Investigational *ERK1/ERK2* inhibitors like Ulixertinib (14) present a potential strategy in cases of resistance due to mutations in *MEK1*, where conventional allosteric *MEK* inhibitors have shown reduced effectiveness (**Figure 4**). Our patient was undergoing evaluation for potential inclusion in a phase 1 study involving Ulixertinib when symptomatic progression of the disease occurred, prompting a shift in approach towards palliative care.

## Conclusion

Approximately 25% of sporadic MTC tumors do not exhibit activating point mutations in either *RET* or *RAS* genes. Analysis of actionable fusion mutations should be considered if there is metastatic disease. High serum calcitonin levels are not pathognomonic for MTC, and tumor histology may overlap with neuroendocrine lung cancers, creating a diagnostic challenge. In the case of metastatic carcinoma, identification of cell of origin can be challenging even with relatively specific makers such as calcitonin. Mutations that induce activation of the *MAP* kinase pathway downstream of *RET* may serve as a potential mechanism of resistance to highly selective *RET* inhibitors.

## Data availability statement

The original contributions presented in the study are included in the article/supplementary material. Further inquiries can be directed to the corresponding author.

## Ethics statement

Written informed consent was obtained from the individual(s) for the publication of any potentially identifiable images or data included in this article.

## Author contributions

RP: Conceptualization, Writing – original draft, Writing – review & editing. PI: Writing – original draft, Writing – review & editing. CG: Writing – original draft, Writing – review & editing. DB: Conceptualization, Writing – original draft, Writing – review & editing.
